# Diaphragmatic Musculophrenic Vessel Injury Following Ultrasound-Guided Pigtail Catheter Insertion: A Rare Case

**DOI:** 10.7759/cureus.100479

**Published:** 2025-12-31

**Authors:** Jenna A Almannaei, Amena F Almubarak, Qasim A Alnahawi, Raja Nadeem, Ghassan Salman

**Affiliations:** 1 General Surgery, King Hamad University Hospital, Muharraq, BHR; 2 Thoracic Surgery, Salmaniya Medical Complex, Manama, BHR

**Keywords:** color doppler ultrasound, diaphragmatic injury, hemothorax, hydropneumothorax, iatrogenic vascular injury, phrenic artery, pigtail catheter, pleural effusion, thoracotomy, ultrasound-guided thoracostomy

## Abstract

Pigtail catheters are widely used as a minimally invasive substitute to large-bore chest tubes for the management of pleural effusion. Although it is considered safe, serious complications may occur. We report a rare case of diaphragmatic musculophrenic artery injury following ultrasound-guided pigtail catheter insertion. A 39-year-old female with multiple comorbidities was admitted with an impression of pleural effusion. She underwent ultrasound-guided pigtail catheter insertion, with drainage of hemorrhagic exudative fluid. Within one hour, she developed severe dyspnea and chest pain. Imaging revealed a hydropneumothorax, necessitating urgent intercostal chest drain placement. Despite initial clinical improvement, she rapidly deteriorated, prompting emergency thoracotomy. Intraoperative findings revealed a bleeding musculophrenic artery at the diaphragmatic surface, likely injured during pigtail placement. Hemostasis was achieved, and the patient recovered uneventfully. This case reports a rare but life-threatening complication of pigtail catheter insertion. Awareness of anatomical proximity to diaphragmatic vessels and prompt recognition of complications are critical to avoid morbidity and mortality.

## Introduction

Pigtail catheters are widely used as a minimally invasive substitute to large-bore chest tubes for the management of pleural effusion and are generally well tolerated by patients. It is a small flexible tube used to drain collections from the pleural space in a minimally invasive approach. Pigtail catheters are inserted under ultrasound or CT guidance and are particularly used in draining pleural fluid with less trauma to tissues and less pain compared to traditional large-bore chest tubes. In a clinical setting, pigtail catheters are used for symptomatic pleural effusions, parapneumonic effusions, malignant pleural effusions, tuberculous effusions, and selected cases of pneumothorax, especially spontaneous or iatrogenic. Patients generally tolerate pigtail catheters well. These catheters allow continuous drainage while improving respiratory symptoms. However, due to their small diameter, they are not suitable for thick pus, clotted blood, or massive hemothorax. Complications are usually minor, including pain at the insertion site, catheter blockage, inadequate drainage, pneumothorax, and infection; however, more serious problems can occur, such as organ injury. Despite these risks, when used in appropriately selected patients and inserted under image guidance, including ultrasound with Doppler to avoid vascular injuries, pigtail catheters may be used for pleural drainage, with success and complications varying according to the clinical context. We report a rare case of musculoskeletal-phrenic artery injury following ultrasound-guided pigtail catheter insertion, highlighting that significant vascular injury can still occur when image guidance is used [1-3].

## Case presentation

**Figure 1:**
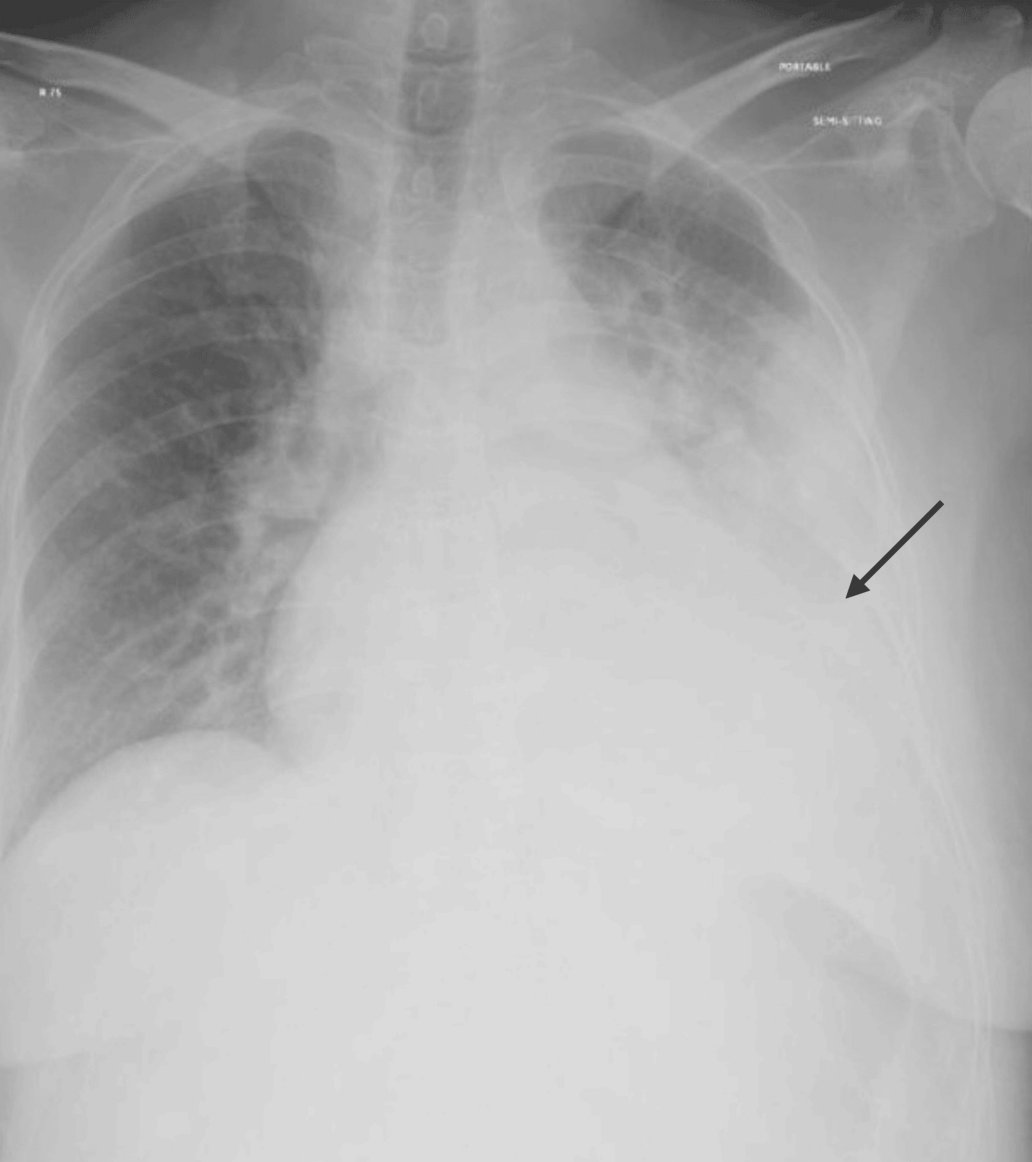
Chest X-ray showing moderate left-sided pleural effusion.

A 39-year-old female with a known diagnosis of multiple myeloma with several medical comorbidities was admitted with an impression of pleural effusion. She was afebrile and maintaining oxygen saturation of 97% on room air. On physical examination, decreased breath sounds were noted at the base of the left lung. Chest X-ray confirmed a moderate left-sided pleural effusion (Figure 1).

Subsequently, she underwent ultrasound-guided pigtail catheter insertion by the interventional radiology team at the level of the left fifth intercostal space, anterior axillary line. Approximately 600 mL of hemorrhagic pleural fluid was drained gradually.

**Figure 2:**
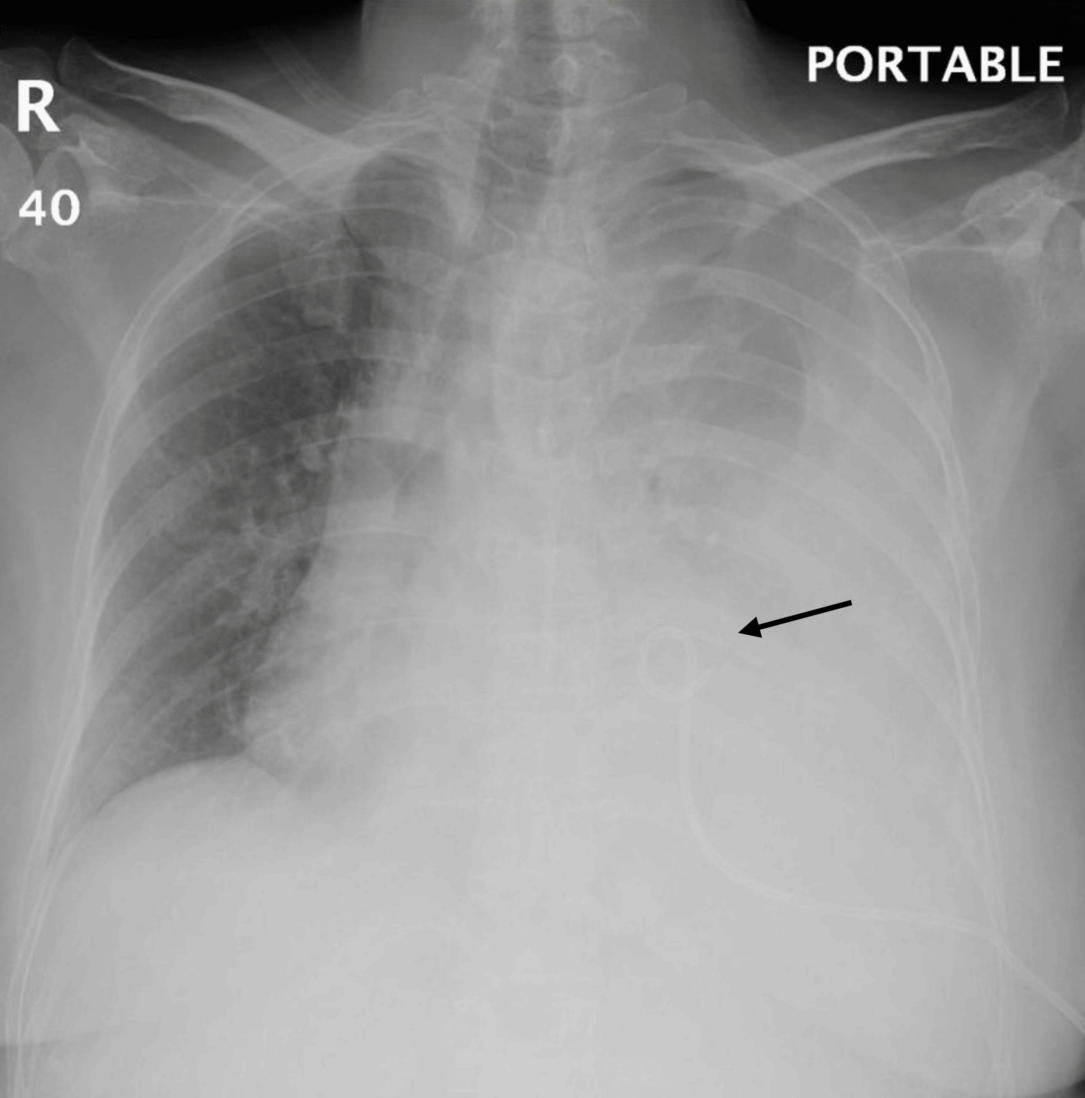
Chest X-ray showing left-sided hydropneumothorax after pigtail insertion.

Within one hour post-insertion, the patient developed sudden-onset dyspnea and severe left-sided chest pain. Vital signs indicated early hemodynamic compromise. Repeated chest X-ray revealed a newly developed pneumothorax with re-accumulation of pleural fluid consistent with a hydropneumothorax (Figure 2).

**Figure 3:**
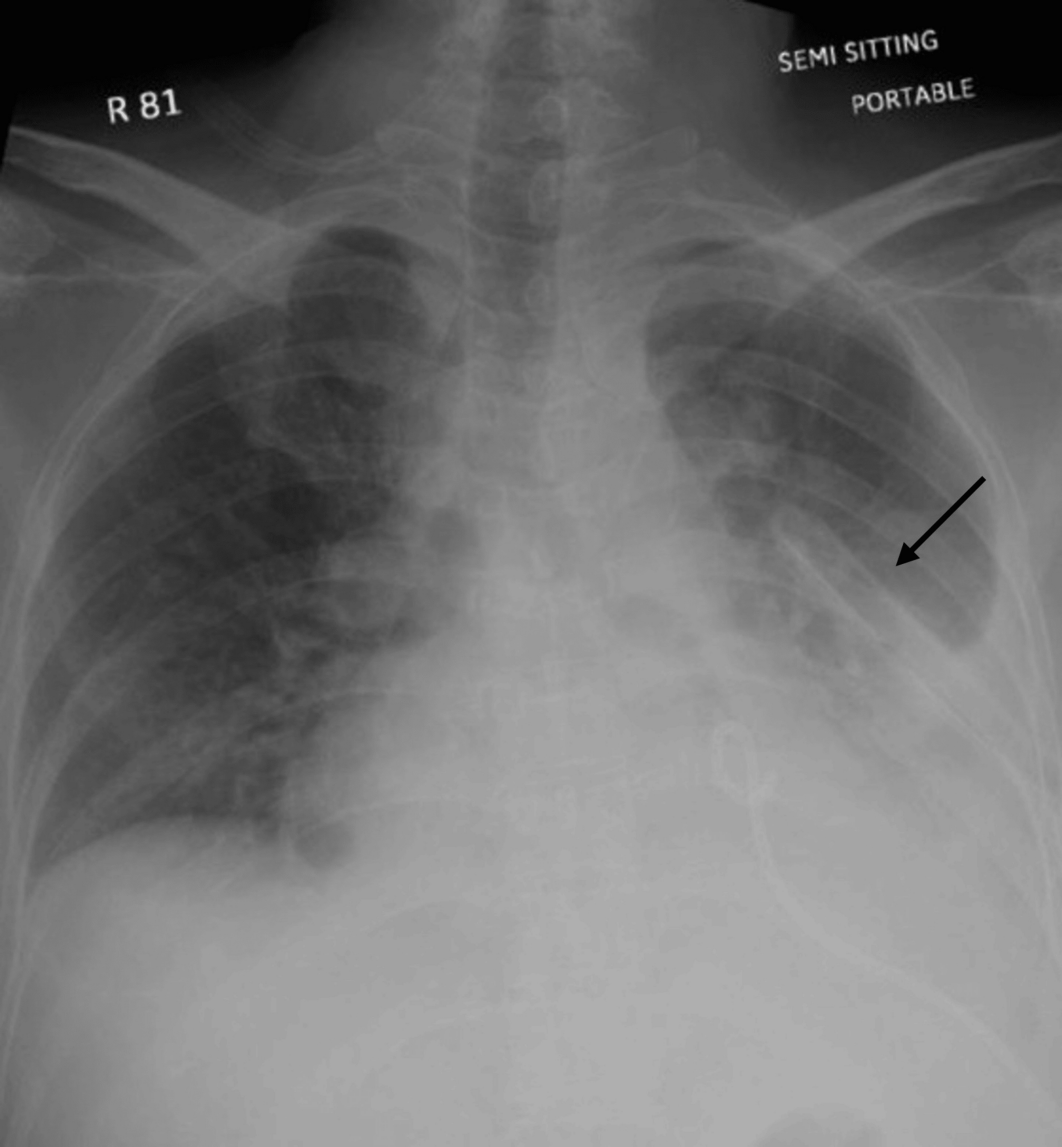
Chest X-ray after the insertion of left-sided intercostal chest drain.

An emergency 28-French intercostal chest drain was placed at the level of the fourth intercostal space mid-axillary line under local anesthesia, with immediate drainage of 1,900 mL of mixed fresh and clotted blood. Chest radiograph confirmed correct positioning of the intercostal chest drain (Figure 3). The patient initially stabilized and showed improvement in symptoms.

**Figure 4:**
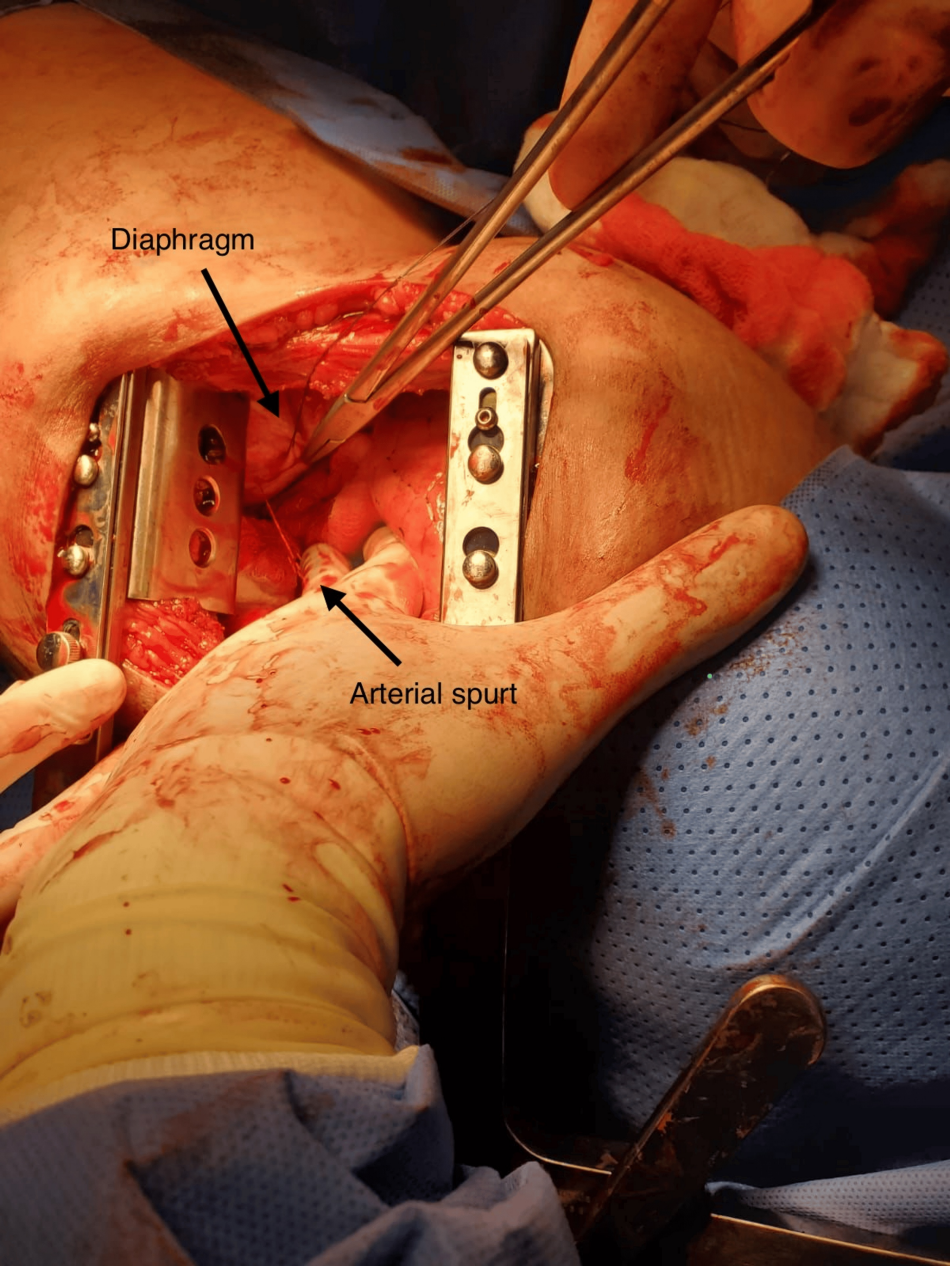
Intraoperative image showing a spurting arterial bleeder on the diaphragmatic surface.

However, approximately 15 minutes later, she experienced acute hemodynamic deterioration. The thoracic surgery team was urgently consulted, and the patient was taken to the operating room for emergency left thoracotomy for bleeding control. Intraoperatively, approximately 600 mL of clotted blood was evacuated. A spurting arterial bleeder was identified on the diaphragmatic surface, suspected to be an injured musculophrenic artery (Figure 4). Hemostasis was achieved surgically.

**Figure 5:**
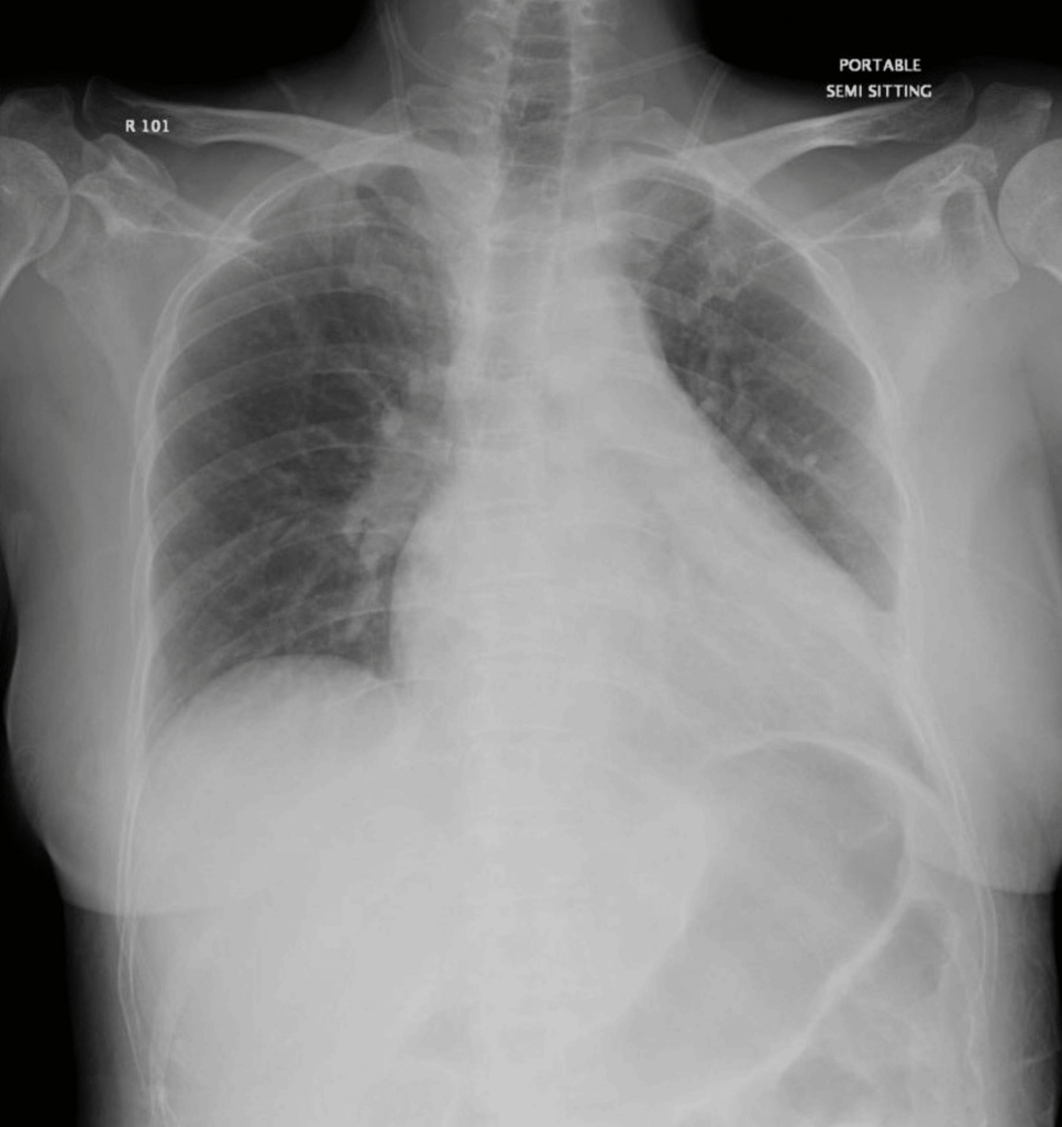
Postoperative chest X-ray showing resolved hemothorax, expanded left lung, and absence of pneumothorax. Chest tube has been removed.

The patient recovered uneventfully postoperatively and was discharged from thoracic surgery care after approximately one week (Figure 5).

## Discussion

Pigtail catheters, due to their small caliber, flexible nature, and enhanced patient comfort, are increasingly used in clinical practice. Despite their overall safety, complications such as pneumothorax, hemothorax, infection, and misplacement may still occur [1-3]. In particular, injuries to intercostal vessels or the diaphragmatic surface are considered exceedingly rare but potentially fatal [2,4]. In our case, the guidewire or catheter most likely penetrated the diaphragm and injured a diaphragmatic intercostal artery, resulting in hemothorax and hemodynamic instability.

Rare cases involving a similar mechanism of injury have been reported. Park and Lee described a case of massive hemothorax due to intercostal arterial bleeding after pigtail removal [4]. Likewise, Casper et al. reported a pseudoaneurysm of the intercostal artery after thoracentesis requiring coil embolization [5]. These reports, along with ours, illustrate the risk posed to vascular structures in the thoracoabdominal junction, especially when insertion is performed close to the diaphragm.

Standard ultrasound-guided thoracostomy significantly reduces complications by enabling visualization of adjacent structures; however, it cannot eliminate them entirely. Studies have shown that using color Doppler ultrasound adds another layer of safety by identifying intercostal vessels, especially when accessing lower intercostal spaces (e.g., below the eighth rib) [1,6,7]. Techniques such as lowering the pulse repetition frequency and adjusting imaging depth improve Doppler sensitivity, enhancing visualization of vascular flow in small intercostal branches [1,7,8]. Unfortunately, color Doppler remains underutilized in bedside procedures despite its demonstrated utility [1,6].

Furthermore, selecting an appropriate insertion site is another critical factor. Intercostal arteries typically run along the inferior margin of each rib and are more prominent and exposed in lower thoracic spaces. Literature suggests that higher intercostal thoracostomies (fourth to sixth spaces) are safer in terms of avoiding major structures [3,9,10]. Although lower intercostal tube insertions (seventh or below) may be necessary to access dependent fluid collections, they carry a higher risk of vascular and diaphragmatic injury [2,9].

In our case, the suspected injury to a diaphragmatic musculophrenic artery was likely caused by guidewire penetration beyond the pleural space into the diaphragm. This rare complication underscores the importance of procedural precision, the potential benefit of color Doppler imaging, and avoiding unnecessarily low punctures without clinical indication.

Prompt identification and immediate surgical intervention were lifesaving in our case. In previously reported cases, both coil embolization and thoracotomy have been successfully employed depending on the patient’s hemodynamic status and severity of bleeding [4,5,9].

It is equally essential to underscore the importance of vigilance during and after placement of the pigtail catheter in the early detection of complications and management accordingly. Confirming pleural entry before inflation of the pigtail balloon by aspirating can add another layer of safety and help prevent accidental penetration of non-pleural structures [11].

Close observation after the procedure remains vital. New symptoms such as chest pain, an increase in bloody output, unstable vital signs, and a sudden drop in hemoglobin level can be early signs of significant complications [12]. A preemptive approach by remaining alert for clinical deterioration and initiating prompt imaging and intervention when required is key to avoiding diagnostic delays and reducing potential harm to the patient [13,14].

## Conclusions

This case highlights a rare but fatal complication following the placement of an ultrasound-guided pigtail catheter, namely, diaphragmatic musculophrenic artery injury. Clinicians should remain vigilant during and after interventions involving the thoracic cavity, particularly when anatomical proximity to major vascular structures exists. Emergency surgical intervention may be required to manage such events and prevent mortality.
